# miR-142-3p Reduces the Size, Migration, and Contractility of Endometrial and Endometriotic Stromal Cells by Targeting Integrin- and Rho GTPase-Related Pathways That Regulate Cytoskeletal Function

**DOI:** 10.3390/biomedicines8080291

**Published:** 2020-08-18

**Authors:** Christin S. Börschel, Anna Stejskalova, Sebastian D. Schäfer, Ludwig Kiesel, Martin Götte

**Affiliations:** 1 Department of Gynecology and Obstetrics, Münster University Hospital, 48149 Münster, Germany; c.boerschel@outlook.com (C.S.B.); SebastianDaniel.Schaefer@ukmuenster.de (S.D.S.); ludwig.kiesel@ukmuenster.de (L.K.); 2 Department of Cardiology, University Heart and Vascular Centre Hamburg-Eppendorf, 20251 Hamburg, Germany

**Keywords:** microRNA, miR-142-3p, endometriosis, cytoskeleton, integrin, collagen, WASL, ITGAV, endometrial stroma cells, in vitro study

## Abstract

Downregulated microRNA-142-3p signaling contributes to the pathogenesis of endometriosis, an invasive disease where the lining of the uterus grows at ectopic locations, by yet incompletely understood mechanisms. Using bioinformatics and in vitro assays, this study identifies cytoskeletal regulation and integrin signaling as two relevant categories of miR-142-3p targets. qPCR revealed that miR-142-3p upregulation in St-T1b cells downregulates Rho-associated protein kinase 2 (*ROCK2*), cofilin 2 (*CFL2)*, Ras-related C3 botulinum toxin substrate 1 (*RAC1*), neural Wiskott-Aldrich syndrome protein (*WASL*), and integrin α-V (*ITGAV*). qPCR and Western-blotting showed miR-142-3p effect on WASL and ITGAV was significant also in primary endometriotic stroma cells. Luciferase reporter assays in ST-T1b cells then confirmed direct regulation of *ITGAV* and *WASL*. On the functional side, miR-142-3p upregulation significantly reduced ST-T1b cell size, the size of vinculin plaques, migration through fibronectin-coated transwell filters, and the ability of ST-T1b and primary endometriotic stroma cells to contract collagen I gels. These results suggest that miR-142-3p has a strong mechanoregulatory effect on endometrial stroma cells and its external administration reduces the invasive endometrial phenotype. Within the limits of an in vitro investigation, our study provides new mechanistic insights into the pathogenesis of endometriosis and provides a perspective for the development of miR-142-3p based drugs for inhibiting invasive growth of endometriotic cells.

## 1. Introduction

MicroRNAs are short non-coding RNAs that post-transcriptionally regulate cellular signaling by dampening the expression of their target mRNAs [1]. This highly conserved class of genes comprises more than 2000 species [2] that constitute around 1% of all predicted genes [1]. MicroRNAs thus provide an important regulatory layer that makes it possible to adjust the dosage of proteins in a cell-type-specific manner and contribute to cells having the appropriate phenotype [1]. Unfortunately, the microRNA regulatory network itself can be affected by mutations [3] or be hijacked to respond to the aberrant signals received from an ectopic niche [4]. It is therefore not surprising that altered microRNA gene expression has been shown to accompany and contribute to several human diseases [3], including those of the human reproductive system [5,6,7].

Endometriosis is a condition where the lining of the uterus grows at ectopic locations, such as the ovaries and the peritoneum, causing severe pain and subfertility [8]. While the exact causes of the disease remain unknown, several studies confirmed that the phenotype of endometrial ectopic cells differs from that found in eutopic (eut) endometrium [5,9,10,11]. For example, ectopic (ect) endometrial cells are progesterone resistant [12], can produce estrogen [13], are contractile [14] and invasive [15]. This could be due to several factors, including altered microRNA expression and its associated post-transcriptional regulation of microRNA targets in the ectopic milieu [16,17,18]. One microRNA gene that is downregulated at ectopic locations and might thus contribute to these phenotypic changes is the microRNA-142-3p [5,19]. Two previous studies on the endometriotic cell line Hs832.Tc (ATCC CRL-7566) [19] and the endometrial stroma cell line ST-T1b [20] identified the transcriptional regulator *KLF9*, the interleukin-6 receptor subunit *IL6ST*/gp130, and steroid sulfatase (*STS*) as regulatory targets of miR-142-3p, however, the exact role microRNA-142-3p plays in the pathophysiology of endometriosis currently remains incompletely understood.

Dysregulated miR-142-3p has been previously implicated in impaired hematopoiesis [21]. More recently, several studies found miR-142-3p to be hypermethylated and downregulated in aggressive metastatic cancers, including pancreatic ductal adenocarcinoma [22], prostate cancer [23], and breast cancer [24], suggesting this microRNA affects cellular invasion across several cell types. Among the regulatory targets of miR-142-3p identified in these non-endometrial tissues and experimental systems were *ADCY9, BOD1, BRCA1, BRCA2, CTNNB/beta-catenin*, *CFL2, FOXO1, IRF7, ITGAV, KLF4, PGRMC2, RAC1, ROCK2*, and *WASL* [20,21,22,23,24,25]. Furthermore, an effect of miR-142-3p on cellular motility was seen in knockout miR-142^−/−^ mice, which exhibited abnormal skin wound healing upon infection [26].

In this study, we use bioinformatics databases to identify the main targets of miRNA-142-3p and their associated pathways, and we validate predicted targets experimentally in both eutopic (uterine) and ectopic endometrial cells using gene expression, protein expression, and functional assays. Our results suggest miR-142-3p contributes to the pathophysiology of endometriosis at ectopic locations by regulating the expression of the Rho GTPases that regulate cytoskeleton and of integrin signaling.

## 2. Methods

### 2.1. Cell Culture

The St-T1b cell line [27] and primary stromal cells (ESCs) derived from ectopic lesions or the eutopic endometrium of endometriosis patients were maintained in 70% DMEM/18% MCDB 105 media (Sigma-Aldrich, Steinheim, Germany, cat. No. 117–500) supplemented with 10% FBS, 1% Pen/Strep, 1% Glutamine, and 5 µg/mL insulin (Sigma-Aldrich, Strinheim, Germany, cat. No. 10516). Cells were routinely split twice a week. ESCs were prepared from ectopic lesions and eutopic endometrium and characterized as previously described [28]. Primary endometriotic stromal cells were prepared from biopsies of women with endometriosis who underwent surgical treatment at the Department of Gynecology and Obstetrics of Münster University Hospital between 2009 and 2010 as well as from October 2012 and March 2014 as described previously [29]. The modified American Society for Reproductive Medicine classification was used to assess endometriosis (ASRM, 1997). The specific cell types derived from ectopic lesions (#1–7) and the eutopic uterine endometrium of endometriosis patients (A,B) are listed in Table A1. The study was carried out following the Declaration of Helsinki and approved by the local ethics commission (Ethikkommission der Ärztekammer Westfalen-Lippe und der Medizinischen Fakultät der WWU; approval no. 1 IX Greb 1 from 19 September 2001, updated 2012). Informed, written consent was obtained from all participants.

### 2.2. microRNA Transfection

The transfection with pre-miR-142-3p, anti-miR-142-3p, or the negative control miRNA #1 (all ABI, Darmstadt, Germany), was performed in a six-well plate on 60–70% confluent cells. To enhance the transfection process, the growth media were exchanged for Opti-MEM I Reduced Serum Media (Gibco^®^, cat. no. 31985-070, Thermo-scientific, Schwerte, Germany) following which a pre-incubated mixture of the microRNA of interest and the Dharmafect reagent (Dharmacon™, Lafayette, CO, USA, cat. no. T-2001-03,) was added to the cells to achieve the final concentration 20 nM microRNA per well. The cells were incubated with the transfection mixture for 24 h following which the Opti-MEM medium was exchanged for the standard serum-supplemented 70% DMEM/18% MCDB 105 medium.

### 2.3. qRT-PCR

Total RNA was extracted 72 h after transfection using the innuPREP RNA Mini Kit (Analytik Jena AG, Jena Germany) according to the manufacturer’s instructions. In total, 1 µg of total RNA was reverse-transcribed using the High-Capacity RNA-to-cDNA kit and microRNA was reverse-transcribed into cDNA using the TaqMan MicroRNA Reverse Transcription Kit (Applied Biosystems, Darmstadt, Germany). RNU6B was used as the reference value. qPCR was performed using the TaqMan Universal PCR Master Mix (Applied Biosystems, Darmstadt, Germany) with 100 ng cDNA per 20 µL. The 18S or RNU6B was used as the reference housekeeping gene. The primers were purchased from Applied Biosystems (Darmstadt, Germany) (*ITGAV* Hs00233808_m1, hsa-mir-142-3p TM 000464, *CFL2* Hs01071313_g1, *RAC1* Hs01902432_s1, *ROCK2* Hs00153074_m1, *WASL* Hs00187614_m1, *RNU6B* TM 001093, and *18S* Hs99999901_s1). The data were analyzed using the delta-delta Ct method [30].

### 2.4. DUAL-Luciferase Reporter Assay

St-T1b cells were seeded in a six-well plate at the concentration 2 × 10^5^ per well and co-transfected with a pre-micro-RNA or anti-microRNA and a plasmid pEZX-MT01-N-WASP(ITGAV)-3′UTR expressing firefly luciferase (hLuc) under the control of an SV40 enhancer and the 3′UTR of human *N-WASP* (*WASL*) (HmiT021768) or *ITGAV* (HmiT055211), and renilla luciferase (hRLuc) under constitutive control of the cytomegalovirus (CMV) promoter (GeneCopoeia, Rockville, MD, USA). Luciferase assays were performed in quadruplets (*n* = 3 experiments) in a 96-well format using the Luc-Pair miR Luciferase Assay (GeneCopoeia, Rockville, MD, USA) exactly as described by the manufacturer. At 72 h after transfection, cell lysates were prepared and assayed in a luminometer normalizing firefly to renilla luciferase activity measured in the same well. Data were expressed as percent inhibition relative to control miR-transfected cells.

### 2.5. Western Blot

The St-T1b cells were lysed 72 h after transfection with 200 µL/well of RIPA-Buffer while the primary endometrial stromal cells were lysed using 150 µL/well SDS-lysis-buffer due to the low protein content. All buffers were supplemented with a proteinase inhibitor cocktail containing NaF, β-glycerol phosphate and NaVO_3_. BCA assay (Pierce, Schwerte, Germany) was used according to the manufacturer’s instructions to analyze the protein concentration of cell lysate. Samples were separated by SDS-polyacrylamide gel electrophoresis under reducing conditions and transferred onto a nitrocellulose membrane and then blocked with 5% BSA–0.1% Tween–20 in PBS. The primary antibodies (ITGAV—polyclonal, rabbit, 1:1000 Cell Signaling, Billerica, MA, USA, N-WASP—monoclonal, rabbit, 1:1000, Cell Signaling, Billerica, MA, USA, Tubulin—monoclonal, mouse, 1:4000, Sigma-Aldrich, Steinheim, Germany) were diluted in blocking solution and incubated with the membranes overnight at 4 °C. The membranes were then washed, and the bands were incubated with the horseradish peroxidase (HRP)-conjugated secondary goat anti-rabbit and goat anti-mouse antibodies (Calbiochem, Darmstadt, Germany) in blocking solution (2.5% skim milk) for 1 h at room temperature. The membranes were developed in the HRP substrate. Tubulin was used in all samples as a reference.

### 2.6. Cell Spread Area Analysis

The cell size was evaluated 56 h after transfection and 8 h after reseeding onto a 24-well-plate at the density of 782 cells/mL. The cells were stained using Crystal-Violet (Sigma-Aldrich, Steinheim, Germany). The cells were imaged at 320-fold magnification using the Axiovert 100 microscope (Zeiss, Oberkochem, Germany) with 15 fields of view per condition. The cells size was evaluated the program Axio Vision 4.0 (Zeiss, Oberkochem, Germany).

### 2.7. Migration Assay

The migration chamber was coated with fibronectin (Becton Dickinson, Franklin Lakes, NY, USA) diluted at 1:100 in PBS. The pore size of the insert was 8 µm. The medium was exchanged 24 h after the transfection. Subsequently, the cells were placed in a medium without FCS and with 0.1% BSA after additional 24 h and seeded on the migration filer in 100 µL suspension and left to migrate for 13 h. Finally, the membrane was fixed in cold methanol (Merck, Darmstadt, Germany) and the cells were stained using Crystal-Violet. The imaging was conducted at 200-fold magnification using the light-microscope Axiovert 100 equipped with the AxioCam MRC camera (Zeiss, Oberkochem, Germany) and the quantification was conducted manually in the program ImageJ (NIH, Bethesda, MD, USA).

### 2.8. Collagen Gel Contraction Assay

The cells were harvested 48 h after transfection. To prepare the hydrogels, transfected cells in media were mixed with collagen I, acid, and distilled water and 500 µL of the mixture per well were pipetted into a 24-well plate to achieve a final collagen I concentration of 2.5 mg/mL and 200,000 cells per well. The hydrogels were imaged using an iPhone 8 camera 48, 96 and 144 h after being prepared. The area was quantified by manually tracing the area of both hydrogels and outer rims of the wells that served as a reference due to their constant area in FIJI (Fiji.sc).

### 2.9. Immunofluorescence Staining

Cells were seeded on round-coverslips coated with 1:100 fibronectin in PBS (overnight, 4 °C) at the concentration 2 × 10^4^ cells/mL (1 mL per six-well plate) 32 h after transfection. The cells were fixed 24 h after seeding with 3.7% Formalin and permeabilized using 0.1% Triton-X-100 and blocked using 1:10 BSA Aurion in PBS. The antibodies were diluted in 1% BSA/PBS solution and stained for 24 h. The vinculin antibody (polyclonal, rabbit, Abcam, Cambridge, UK) was diluted at 1:300. Each coverslip was then washed three times with PBS. The secondary antibody goat-anti-rabbit IgG Alexa Fluor^®^ 488 (1:500, Invitrogen, Eugene, OR, USA) was diluted 1:500 in 1% BSA/PBS, and Alexa Fluor 568 Phalloidin was used to stain F-actin at 1:1000. The nuclei were stained using DAPI (1:2000) in 1% BSA/PBS for 5 min.

### 2.10. Imaging

Imaging was conducted using 630-magnification (five images per coverslip) using the Fluorescence microscope Axioskop 2 equipped with the AxioCam HRC Camera (Zeiss, Oberkochem, Germany). (Green and red—800 ms, DAPI—40 ms). Adobe Photoshop Elements 7.0 (Adobe, San Jose, CA, USA) and Inkscape 0.92v (inkscape.org) were used to assemble the images.

### 2.11. Bioinformatics

The target genes were predicted using miRTarget, Diana and TargetScan. For the miRTarget database, only the predicted mRNAs with the Target Score 81–100 were considered as the most likely to be real and consequently, out of the total of 418 predicted targets, only 169 that satisfied this condition were considered for further analysis. The target genes predicted by all three databases were identified using the Venn diagram powered by the http://genevenn.sourceforge.net software. The pathways in which the predicted genes play a role was subsequently predicted using the PANTHER website (http://www.pantherdb.org).

### 2.12. Statistical Analysis

Data were analyzed using GraphPad Prism8 (GraphPad Software, San Diego, CA, USA). Normal distribution was tested using the Shapiro-Wilk test. Two-tailed unpaired Student’s *t*-tests were used to analyze statistical significance between two conditions in an experiment. For experiments with three or more comparisons, an ordinary one-way ANOVA with a Tukey’s multiple comparisons test was used. For data that were not normally distributed, the Kruskal-Wallis test followed by Dunn’s multiple comparisons test was used. A two-way repeated-measures (RM) ANOVA with Šidák’s multiple comparisons test was used to evaluate the effect of Matrigel and collagen I on spheroid size over time. Significance values were chosen as * *p* < 0.05; ** *p* < 0.01; *** *p* < 0.001, **** *p* < 0.0001 and not significant (n.s.) as *p* > 0.05. Error bars represent the mean ± standard deviation (s.d.) or mean + s.d. All figure panels were assembled in Inkscape 0.92 (inkscape.org).

## 3. Results

### 3.1. In Silico Analysis Predicts miRNA-142-3p Targets Several Rho GTPases and Integrin Signaling Pathway Constituents

**Figure 1:**
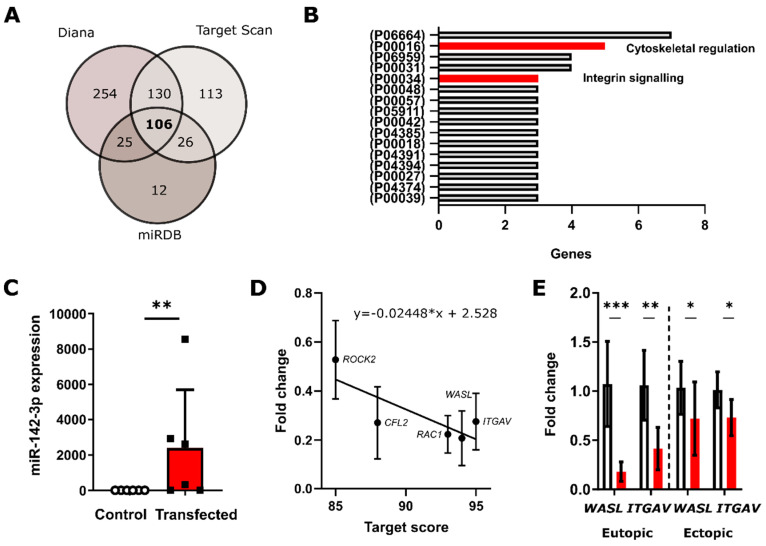
miR-142-3p targets multiple genes involved in the cytoskeletal regulation and integrin signaling pathways (**A**) Venn diagram depicting mRNA targets of miR-142-3p predicted by three independent algorithms Diana, Target Scan, and miRDB. (**B**) Pathways predicted by PANTHER that contain three or more predicted target genes of miR-142-3p. The codes can be looked up in the panther database. (**C**) qPCR analysis of miR-142-3p transfected St-T1b cells (*n* = 6, *t*-test). (**D**) qPCR identified that the predicted gene products *ROCK2*, *CFL2*, *RAC1*, *WASL*, and *ITGAV* are significantly downregulated in St-T1b compared to controls (*n* = 3, *t*-test) and that the degree to which they are downregulated correlates with the predictions of the miRDB algorithm (y = −0.02448*x + 2.528). (**E**) The targets *WASL* and *ITGAV* are downregulated by miR-142-3p both in eutopic (eut) (uterine) and ectopic (ect) primary endometrial stromal cells (ESCs) white bars represent control cells and red bars transfected cells (*WASL*: *p*_eut_ = 0.0006, *p*_ect_ *=* 0.0277, *n*_eut_ *=* 6, *n*_ect_ *=* 13, *ITGAV: p*_eut_ *=* 0.0035, *p*_ect_ *=* 0.0234, *n* = 6, *t*-test). Data in the whole figure panel represent mean ± s.d. For all figures in this panel * *p* < 0.05; ** *p* < 0.01; *** *p* < 0.001, and ^n.s.^ *p* > 0.05.

The putative mRNAs targeted by miR142-3p were identified in silico using a conservative approach that relies on three algorithms MirTarget [31] (miRDB.org), Diana, and TargetScan. In total, 106 target genes were predicted by all three algorithms and were thus considered as the likely targets of miR-142-3p (Figure 1A). The functions and the signaling pathways these mRNAs are involved in were assessed using the PANTHER classification system [32]. The predicted targets were present in a total of 61 pathways. Figure 1B lists the 16 pathways in which three and more targets are regulated by miR-142-3p, as it can be assumed that the more members of a particular pathway are targeted, the more profound the phenotypic effect of a given microRNA will be. In this study, we specifically focused on the mechanoregulatory pathways *Cytoskeletal regulation by Rho GTPase* (P00016) and the *Integrin Signaling Pathway* (P00057). Additionally, miR-142-3p was predicted to target seven members from *the gonadotropin-releasing hormone receptor* pathways, four from *CCKR signaling map and inflammation mediated by chemokine and cytokine signaling* pathway and three from the *PI3 kinase and Wnt signaling* pathways. The specific predicted miR-142-3p targets from the *Rho GTPase* (P00016) pathway were *Ras-related C3 Botulinum toxin substrate 1 (RAC1), Myosin light chain (Myosin), Cofilin (CFL2), Rho-associated coiled-coil-containing protein kinase (ROCK2)* and *Wiskott-Aldrich syndrome protein family member 1 (WASL)* and from the *Integrin Signaling* Pathway (P00057) *RAC1, Integrin beta-8 (ITGB8),* and *Integrin alpha-V (ITGAV)*.

### 3.2. qRT-PCR Validates miR-142-3p Regulates ROCK2, CFL2, RAC1 and WASL Gene Expression Levels

To confirm the regulation of predicted targets of miR-142-3p in vitro, we employed a transient transfection approach in the immortalized human endometrial stroma cell line St-T1b [27] and primary eutopic (uterine) and ectopic endometrial stromal cells of endometriosis patients. MicroRNA transfection significantly increased (*p* = 0.0022, *n* = 6) the levels of intracellular miR-142-3p with a median fold microRNA increase of 1467 (Figure 1C). qPCR confirmed that the levels of the predicted mRNA targets from the *Cytoskeletal regulation by Rho GTPase* pathway (P00016) *ROCK2* (*p* = 0.029736, *n* = 3), *CFL2* (*p* = 0.00375, *n* = 3), *RAC1* (*p* = 0.000455, *n* = 3), and *WASL* (*p* = 0.001383, *n* = 3) and of the member of the *Integrin signaling* pathway (P00034) *ITGAV* (*p* = 0.001663, *n* = 3) were significantly reduced in St-T1b cells upon transfection with miR-142-3p (Figure 1D). The fold change of repression was inversely correlated (y = −0.02448*x + 2.528) with the predicted Target Score from the miRDB algorithm. We further validated that miR-142-3p also significantly affects *WASL* (*p*_eut_ = 0.0006, *n*_eut_ = 6, *p*_ect_ = 0.0277, *n*_ect_ = 13) and *ITGAV* (*p*_eut_ = 0.0035, *n*_eut_ = 6, *p*_ect_ = 0.0234, *n*_ect_ = 6) expression in primary eutopic and ectopic endometrial cells (ESCs) (Figure 1E).

### 3.3. Luciferase Validation of The Predicted Targets *WASL* and *ITGAV*

**Figure 2:**
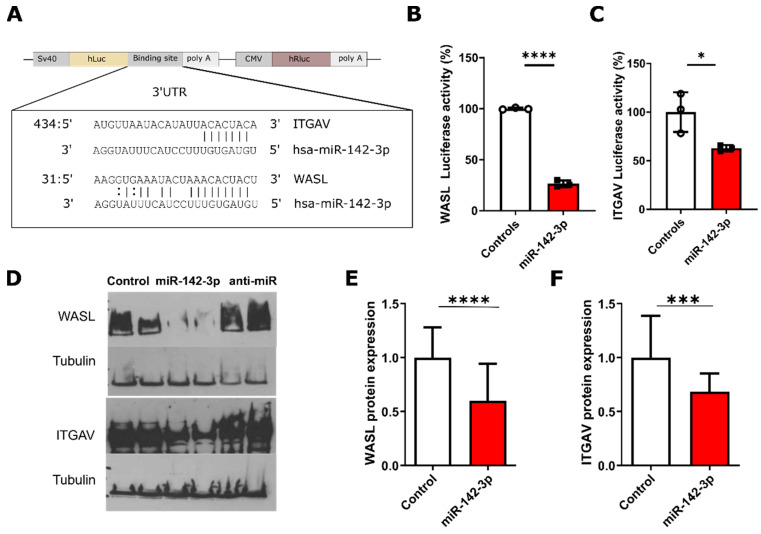
miR-142-3p inhibits WASL and ITGAV protein expression at the post-transcriptional level. (**A**) Luciferase 3′UTR reporter vectors were used to assess hsa-miR-142-3p binding to the 3′UTR of *ITGAV* and *WASL* mRNA. The insert shows complementary miR-142-34p targeting regions of the 3′UTRs of *ITGAV* and *WASL* derived from the microRNA.org database. (**B**) miR-142-3p significantly (*p* < 0.0001, *n* = 3, *t*-test) inhibits *WASL*-dependent luciferase activity. (**C**) miR-142-3p inhibits *ITGAV*-dependent luciferase activity (*p* = 0.0359 *n* = 3, *t*-test). (**D**) Western blot analysis shows that miR-142-3p reduces WASL and ITGAV protein levels in St-T1b cells, while anti-miR has no clear effect. (**E**) miR-142-3p reduces the level of WASL protein expression in primary ectopic ESCs (*p* < 0.0001, *n* = 24, *t*-test). (**F**) miR-142-3p reduces the level of ITGAV protein expression in primary ectopic ESCs (*p* = 0.0019, *n* = 19, *t*-test). Data in the figure panel represent mean ± s.d or mean + s.d. For all figures in this panel * *p* < 0.05; *** *p* < 0.001, **** *p* < 0.0001 and ^n.s.^ *p* > 0.05.

The predicted miR-142-3p targets *WASL* and *ITGAV* were validated using the reporter Dual-Luciferase reporter assay, where the 3′-UTR of these genes were cloned into plasmids downstream of the luciferase reporter proteins (Figure 2A), thus allowing to test if miR-142-3p function depends on a direct interaction with the 3′UTR of the predicted target mRNA. In both cases, miR-142-3p significantly (*p_WASL_* < 0.0001, *p_ITG__AV_* = 0.0359, *n* = 3) reduced reporter luminescence, confirming these genes as direct miR-142-3p targets (Figure 2B,C).

### 3.4. Protein-Level Validation of *WASL* and *ITGAV*

Western blot revealed that miR-142-3p regulates *WASL* and *ITGAV* protein levels both in St-T1b and primary ectopic ESCs. Figure 2D shows that the transfection with miR-142-3p significantly decreased *WASL* (*p* = 0.0258, *n* = 6) and *ITGAV* (*p* = 0.0008, *n* = 6) protein levels in St-T1b, while anti-miR-142-3p had no significant effect on the levels of these proteins. This was also the case in primary ectopic ESCs obtained from six patients where miR-142-3p significantly inhibited *WASL* (*p* < 0.0001, *n* = 24) (Figure 2E) and *ITGAV* (*p* = 0.0019, *n* = 19) translation (Figure 2F).

### 3.5. miR-142-3p Lowers the Concentration of Vinculin in Focal Adhesions

**Figure 3:**
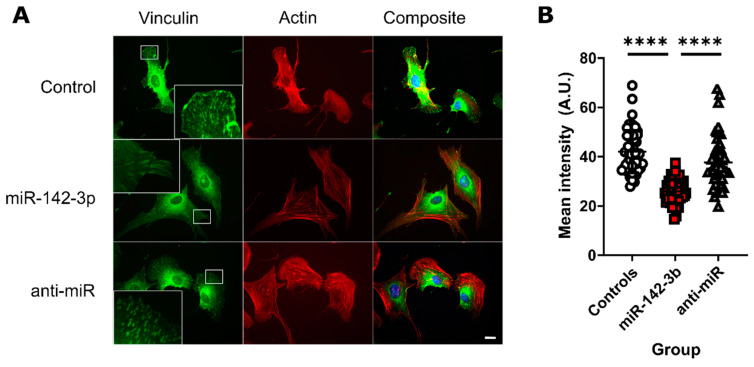
Vinculin plaques are less prominent in miR-142-3p-transfected cells. Green fluorescent staining represents vinculin, whereas red fluorescent staining shows fluorescently labelled phalloidin binding to actin filaments. (**A**) There are fewer vinculin plaques and the plaques have lower green fluorescence intensity compared with the controls and anti-miR transfected St-T1b cells. Vinculin also localized to the endoplasmic reticulum and the Golgi apparatus. Scale bar, 10 µm. (**B**) Quantification shows that the intensity of vinculin in focal adhesions is reduced after transfection with miR-142-3p (*n* = 43–54, Kruskal-Wallis test). Data in the whole figure panel represent mean ± s.d. For all figures in this panel **** *p* < 0.0001 and ^n.s.^ *p* > 0.05.

We investigated the effect of miR-142-3p on the formation of focal adhesions. To visualize the focal adhesion plaques, we stained the membrane-cytoskeletal protein vinculin, one of the components of focal adhesion complexes [33]. There was a smaller number of identifiable vinculin plaques in the miR-142-3p transfected cell compared to other conditions (Figure 3A). Quantitative analysis revealed that the mean intensity of individual vinculin plaques was significantly lower in miR-142-3p transfected cells compared to both scrambled controls (*p* < 0.0001, *n* = 43–54), and anti-miR-142-3p transfected cells (*p* < 0.0001, *n* = 43–54) (Figure 3B). Apart from focal adhesions, vinculin was visible in the endoplasmic reticulum and the Golgi apparatus, probably reflecting transport of vinculin through the secretory pathway (Figure 3A).

### 3.6. Functional Analysis of the Role of miRNA-142-3p in Endometriosis Suggests Its Effects on Endometrial Stromal Cell Size, Migration, and Collagen I Contractility

**Figure 4:**
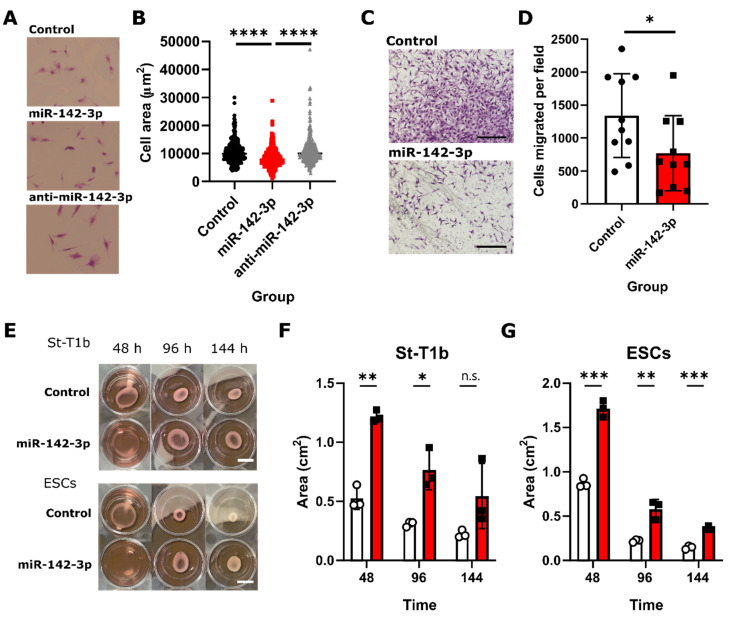
miR-142-3p affects cell size, invasion and contractility in St-T1b cells. (**A**) MiR-142-3p reduces stromal cell size, 320-fold magnification. (**B**) Quantification of the effect of miR-142-3p on stromal cell size (*n* = 376, Kruskal-Wallis test). (**C**) Migration assay through fibronectin (an ITGAV ligand)-coated chamber. Scale bar, 250 µm. (**D**) MiR-142-3p reduces the migratory potential of stromal cells (*n* = 10, *t*-test). (**E**) Collagen contraction assay after 48, 96, and 144 h in St-T1b and ectopic stromal cells (ESCs), Scale bar, ~5 mm. (**F**,**G**) MiR-142-3p reduces contractility of both eutopic St-T1b and ectopic ESCs cells (*n* = 3, multiple *t*-tests, data are representative of two independent repeats). Data in the whole figure panel represent mean ± s.d. For all figures in this panel * *p* < 0.05; ** *p* < 0.01; *** *p* < 0.001, **** *p* < 0.0001, and ^n.s.^ *p* > 0.05.

We investigated how miR-142-3p affects the St-T1b and ESC phenotype. MiR-142-3p treatment resulted in a significantly smaller (*p* < 0.0001, *n* = 376) projected area in St-T1b cells while anti-miR had no significant effect (*p* > 0.999, *n* = 376) (Figure 4A,B). The mean projected areas were 10,493 ± 3866, 8175 ± 3400, and 10,955 ± 4774 µm^2^ for the control, miR-142-3p, and anti-miR-142-3p groups, respectively. Further, we assessed the effect of miR-142-3p on migration through filter membranes coated with fibronectin, an *ITGAV*-ligand [34]. Our results (Figure 4C,D) show that miR-142-3p significantly reduced (*p* = 0.0486, *n* = 10) the number of migratory cells per field in ectopic cells from 1340 ± 636 to 770 ± 568 per field. Finally, we employed the collagen I hydrogel contraction assay to evaluate the functional effect of miR-142-3p on the actin cytoskeleton. Figure 4E–G shows that miR-142-3p significantly reduced the ability of endometrial stromal cells to contract collagen in both St-T1b and primary ectopic stromal cells after 48 h (*p*_St-T1b_ ** = 0.001195, *p*_ESC_ *** = 0.00049, *n* = 3) and 96 h (*p*_St-T1b_ * = 0.018911, *p*_ESC_ ** = 0.004557, *n* = 3). The effect of a single transfection lasted around 144 h until it got partially lost (*p*_St-T1b_^n.s.^ = 0.115764, *p*_ESC_ *** = 0.00056, *n* = 3).

## 4. Discussion

Endometriosis is an invasive disease where the ectopic lesions exhibit aberrant gene expression and epigenetic regulation compared to the eutopic endometrium [5,35]. One downregulated gene in the ectopic lesions is miR-142-3p [5]. In this study, we used a combination of in silico target prediction and experimental validation to evaluate the role of microRNA-142-3p in the pathophysiology of endometriosis.

Each microRNA regulates tens to hundreds of targets [1]. To aid the discovery of these targets, several computational approaches with advantages and disadvantages each have been developed [36]. To minimize the number of false-positive findings, we conducted a conservative computational analysis using MirTarget [31], TargetScan, and Diana and considered only the 106 genes that were predicted by all three algorithms for further analysis. We then performed a gene ontology (GO) analysis, because microRNAs targeting multiple mRNAs from one pathway might hereby regulate cellular phenotype [37]. Our analysis predicted that miR-142-3p targets five members of *Cytoskeletal regulation by Rho GTPase* (P00016) and three members of the *Integrin Signalling* (P00034) pathways, suggesting that miR-142-3p may contribute to the pathogenesis of endometriosis to a big extent through targeting the mechanoregulatory apparatus, which may affect invasive growth at ectopic locations.

We experimentally validated the predicted miR-142-3p targets *ROCK2, CFL2*, *RAC1*, *WASL*, and *ITGAV* in St-T1b cells using qPCR, which is in accordance with our previous findings in breast cancer cells [24]. Regarding the mandatory miR-142-3p-expression-level leading to regulatory effects, our results are in agreement with predictions by a machine-learning miRTarget algorithm [36]. Furthermore, we used Dual-Luciferase assay and Western-blotting to confirm that miR-142-3p downregulates *WASL* and *ITGAV*. *WASL* is a scaffolding protein activated by Cdc42 that stimulates actin polymerization, is involved in filopodia formation [38], and plays a critical role in endocytosis and phagocytosis [39]. *ITGAV* is an integrin, that mediates binding to fibronectin, vitronectin, fibrinogen and is upregulated in women with endometriosis when compared to healthy women during menstruation [40]. *ITGAV* might thus contribute to the increased ability of endometrial cells and tissue fragments to adhere and migrate at ectopic sites during the retrograde menstruation. In breast cancer cells, either siRNA-depletion of *WASL* or miR-142-3p upregulation resulted in reduced formation of proinvasive membrane protrusions, whereas siRNA knockdown of either *WASL* or *ITGAV*, or miR-142-3p upregulation inhibited invasive growth in vitro [24].

In contrast to the previously described upregulation of the miR-142-3p target *STS* by antimiR-142-3p treatment of St-T1b cells [20], antimiR-treatment did not significantly affect expression of the targets described in the present study, and of *IL6 ST* in our previous work [20]. We speculate that the upregulation of a miRNA that is expressed at low levels in a given cell type is more effective that its expression with respect to target regulation.

The collective targeting of multiple cytoskeletal and integrin genes such as *ITGAV* and *WASL* by miR-142-3p indirectly affected the assembly of focal adhesion complexes, which we visualized by staining the focal adhesion protein vinculin [33]. While none of the algorithms predicted vinculin to be a direct target of miR-142-3p, the smaller area occupied by vinculin plaques is not surprising given that the assembly and stabilization of focal adhesion complexes require small *GTPases Rho and RAC* [41] and integrins [42], which we confirmed as miR-142-3p targets.

Our results also revealed that miR-142-3p reduces endometrial stromal cell size, cellular ability to migrate through fibronectin-coated insert and contract collagen I hydrogels. Interestingly, despite the strong effects of miR-142-3p in our experiments, anti-miR-142-3p had no significant effect. This could be due to already low basal levels of this microRNA [43] in endometrial stromal cells [5]. The described effects of miR-142-3p might be similar across more cell types, as our laboratory has previously shown that miR-142-3p also decreases cell area and migration in breast cancer cell lines [24]. Similar effects of miR-142-3p on migration in endometrial stromal cells were previously observed by Ma and colleagues [19], who attributed these observations to the miR-142-3p effect on *KLF9* and *VEGFA* expression. However, none of our used databases predicted *KLF9* as a miR-142-3p target. Our data support the alternative and complementary explanation that miR-142-3p affects these processes by targeting the cytoskeletal and integrin apparatus. This assumption is in accordance with previous studies indicating an independent effect of *ROCK*, *RAC1* [44], and *cofilin* [45] on cellular contraction and migration.

Overall, our study reveals a strong mechanoregulatory effect of miR-142-3p in endometrial stromal cells. Indeed, the ability of miR-142-3p to regulate invasive behavior has been previously demonstrated in breast cancer [24], hepatocellular carcinoma [46], and immune cells [26,47]. However, this study is the first to demonstrate the mechanoregulatory effects of miR-142-3p in non-cancerous and non-immune endometrial cell type and thus supports the notion that a dysregulated miR-142-3p signaling contributes to the pathophysiology of invasive cellular phenotypes.

MicroRNAs have a multifactorial effect and alternative approaches by which miR-142-3p contributes to the pathogenesis of endometriosis should be further explored under various conditions. For example, our group has previously demonstrated that miR-142-3p targets *STS* and interleukin-6-coreceptor *gp130* in endometrial stroma cells [20] indicating a possible relevance for dysregulation of the endocrine milieu and chronic inflammation in endometriosis. Future studies should, in particular, explore the role of other important pathways predicted to be targeted by miR-142-3p in silico that were previously implicated in endometriosis, including the *gonadotropin-releasing hormone receptor pathway*, *CCKR signaling map, inflammation mediated by chemokine and cytokine signaling pathway*, the *PI3 kinase* and *Wnt signaling pathway*.

While our findings identify an important mechanoregulatory role for miR-142-3p in endometriosis, our in vitro approach has limitations. Our study was performed in vitro on stromal cells only, which does not reflect the more complex composition of the endometrium in vivo, where glandular epithelial cells, infiltrating leukocytes, and vascular cells may be affected in a different manner by miR-142-3p. Moreover, as our previous work and work by others has demonstrated [19,20], the targets identified in this study are not the only molecules regulated by miR-142-3p. Therefore, additional targets may have contributed to the functional impact of this miRNA in our functional assay. Finally, wound healing studies in miR-142-3p mice [26] and retroviral delivery studies in an in vivo model of endometriosis [19] suggest that confirmation of our findings in more complex in vivo models of endometriosis may be worthwhile.

## 5. Conclusions

Taken together, our study extends previous findings that miR-142-3p reduces the pro-migratory and contractile endometriotic phenotype and provides novel insights into the molecular mechanoregulatory mechanisms by which miR-142-3p likely exerts these effects. Finally, our study provides evidence that targeted delivery of this microRNA could be explored as a therapeutic strategy for endometriosis.

## Data Availability

All data generated for the manuscript has been included in the study and are available upon request.

## Appendix A

**Table A1:** List of donors. #1–7 = ectopic lesions; A,B = eutopic (uterine) endometrium of endometriosis patients.

Number	Patient Age at Biopsy	rASRM Score	Endometriosis Manifestations	Location of the Biopsy
#1	33	III	Plica vesicouterina, Lig. Sacrouterinum right. Pelvic wall left, Ovary left	Plica vesicouterina
#2	35	IV	Septum rectovaginale. Pelvic wall both sites. Douglas space	Septum rectovaginale
#3	39	III	Uterus, Ovary both sites, Pelvic wall both sites, Bladder peritoneum, Douglas space	Uterus
#4	33	II	Plica vesicouterina, Pelvic wall left, Lig. Sacrouterinum both sites, Douglas space, Rectum	Plica vesicouterina
#5	19	III	Pelvic peritoneum both sites, Douglas space, Septum rectovaginale, Vagina	Pelvic peritoneum
#6	22	III	Pelvic wall left, Ligamentum sacrouterinum left, Rectum	Pelvic wall, left
#7	39	III	Peritoneum, bladder, pelvic wall close to the urethra	Peritoneum
#A	30	III	Pelvic wall left, Lig. Sacrouterinum left, Plica vesicouterina, Ovary both sides, Recessus ovarian both sites, Douglas space. Rectal front wall	Eutopic uterine endometrium
#B	37	III	Pelvic wall both sides, Abdominal front wall left. Ovary right. Douglas space, Rectum	Eutopic uterine endometrium
